# Longitudinal changes in anatomic biomarkers on optical coherence tomography angiography in diabetic retinopathy

**DOI:** 10.3389/fopht.2026.1860036

**Published:** 2026-07-09

**Authors:** Rachel Liu, Isaac Bakis, Megan Steinkerchner, Kevin Sun, Sapna Gangaputra, Stephen J. Kim, Lok Hin Lee

**Affiliations:** 1 Vanderbilt University School of Medicine, Nashville, TN, United States; 2 Vanderbilt Eye Institute, Nashville, TN, United States

**Keywords:** biomarker, diabetic retinopathy, nonproliferative diabetic retinopathy, OCTA, proliferative diabetic retinopathy

## Abstract

**Purpose:**

To identify optical coherence tomography angiography (OCTA)-derived biomarkers correlating with diabetic retinopathy (DR) severity and characterize longitudinal retinal microvascular changes across DR stages.

**Methods:**

In this 3-year prospective study, we analyzed OCTA images from 328 eyes of 164 adults with type II diabetes and 33 eyes from 17 healthy controls. Patients were categorized as no DR, mild, moderate, or severe nonproliferative DR (NPDR), or proliferative DR (PDR) at baseline. Bilateral OCTA scans were obtained at each visit and processed with a validated pipeline to extract seven microvascular indices from the superficial and deep capillary plexuses: vessel density, skeleton density, acircularity index, average vessel caliber, foveal avascular zone (FAZ) area, FAZ perimeter, and fractal dimensions. Linear regression quantified changes over time, and intergroup comparisons were made using ANOVA and *post-hoc* testing.

**Results:**

A total of 361 eyes (328 diabetic eyes and 33 control eyes) were followed for a mean of 3 years. Longitudinal analysis demonstrated stage-dependent microvascular changes across DR severity groups. In the deep capillary plexus, eyes with severe NPDR exhibited greater annual declines in vessel density (-0.563 ± 0.39% per year) and skeleton density (-0.296 ± 0.23% per year) compared with controls. Acircularity index increased over time, with greater annual increases observed in severe NPDR relative to mild NPDR (0.16 ± 0.12% per year), consistent with progressive macular ischemia. In the superficial plexus, eyes with severe NPDR showed greater annual reductions in average vessel caliber (-4.5e-4 ± 0.0005% per year) compared with earlier disease stages. Across the cohort, most OCTA metrics demonstrated small but statistically significant longitudinal trends, reflecting gradual microvascular remodeling detectable over repeated measurements.

**Conclusions:**

Longitudinal OCTA analysis reveals stage-specific microvascular changes in DR. These dynamic biomarkers may provide early indicators of DR progression and offer a quantitative foundation for individualized monitoring and timely intervention strategies.

**Translational Relevance:**

OCTA-derived biomarkers offer a noninvasive tool for detecting early DR changes and guiding personalized care strategies.

## Introduction

Diabetic retinopathy (DR) is characterized by microvascular ischemia leading to progressive damage to the retinal microvasculature and retinal neurodegeneration, processes that can occur in parallel and whose relative timing may vary between patients (1–4). More recently, optical coherence tomography angiography (OCTA) has emerged as an alternative to invasive and time-intensive fundus fluorescein angiogram (FFA) and provides high-resolution images of both the deep and superficial capillary plexuses of the retina (5, 6).

Several studies have demonstrated the utility of OCTA in characterizing the severity of DR using microvascular parameters. Lee et al. demonstrated that vessel tortuosity increased with the severity of non-proliferative DR (NPDR) but decreased in proliferative DR (PDR), suggesting alterations in vascular remodeling during the progression of DR (7). Sun et al. revealed that OCTA parameters like foveal avascular zone (FAZ) area, vessel density, and fractal dimension were significantly associated with DR progression and the development of diabetic macular edema (DME) (8).

Despite these promising findings, there are limitations in the current body of literature. Most studies on OCTA and DR have been cross-sectional, providing only a snapshot of microvascular changes at a single point in time (7, 9). While these studies have established correlations between OCTA parameters and DR severity, they are unable to provide insight into how these parameters change longitudinally as the disease progresses or regresses in response to treatment. Current longitudinal studies on OCTA and DR are limited in breadth and scope, often including only one stage of DR or only one microvasculature metric (8, 10). Additionally, there is a lack of standardization in OCTA acquisition protocols and quantitative metrics across studies, making direct comparisons difficult.

Our study aims to longitudinally assess changes in retinal microvascular indices on OCTA across different stages of DR. We aim to characterize OCTA biomarkers that correlate with DR severity. This will improve the clinical utility of OCTA in monitoring the changes in DR over time and detecting pre-clinical changes in retinal microvasculature.

## Materials and methods

### Study design and population

This study utilized OCTA images collected during the Inflammatory Mediators in the Pathophysiology of Diabetic Retinopathy (INSPIRE) trial, a single-center prospective 3-year randomized clinical trial conducted at Vanderbilt Eye Institute which enrolled 328 eyes of 164 adult patients with type II diabetes (11). Participants were initially categorized into no DR, NPDR, and PDR based on the International Clinical Disease Severity Scale. Patients in the NPDR cohort were further divided into mild NPDR, moderate NPDR, and severe NPDR based on clinician assessment of fundus findings (11). DR severity was graded once at baseline and was not re-graded longitudinally during follow-up. All diabetic patients underwent complete ophthalmic examination including wide-field fundus photography and optical coherence tomography (OCT) and OCT-angiography every 4 months for 3 years, with 5 repeat scans of the same eye at each visit. All OCTA images were acquired using the Optovue Avanti with a 5x5 macular scan protocol. 33 eyes from 17 healthy control patients underwent yearly OCTA imaging. Best-corrected visual acuity was measured using Early Treatment Diabetic Retinopathy Study (ETDRS) charts and converted to logarithm of the minimum angle of resolution (logMAR) units for analysis. All patients received anti-VEGF or PRP treatments consistent with standard clinical care throughout the duration of this study. The study adhered to ethical guidelines, receiving approval from the Vanderbilt University Medical Center Institutional Review Board (#201433), and all patients provided written informed consent. It complied with HIPAA regulations and the Declaration of Helsinki, with trial registration at ClinicalTrials.gov (NCT04505566) and funding from the National Eye Institute (R01-EY031315).

### Analysis of optical coherence tomography angiography

Retinal vessel metrics were extracted using a systematic image-processing pipeline applied to en-face OCT images that has previously been reported and validated (12, 13). Each image underwent a top-hat morphological filtering step to enhance vessel contrast and diminish background intensity fluctuations. This was followed by a Hessian-based filter designed to highlight tubular structures by emphasizing their second-order intensity patterns. To create a preliminary vessel map, the resulting output was subjected to Huang’s entropy-based thresholding, which provides an adaptive global threshold. In parallel, a local median adaptive thresholding procedure was independently conducted on the preprocessed OCTA images. The two threshold images were then merged by selecting the pixels common to both, to create a vessel segmentation map. Finally, the resulting vessel map was skeletonized, reducing each vessel to a single-pixel-wide representation suitable for downstream quantitative analyses.

FAZ segmentation was performed using a morphological Chan-Vese (MCV) active contour approach (12–17). The original OCTA images were first smoothed with a Gaussian filter to reduce high-frequency noise and improve edge definition. A circular initial level set was positioned near the geometric center of each image to guide the MCV model’s iterative evolution toward the anatomical location of the FAZ. Following convergence, a binary mask of the FAZ was generated and further refined by filling any small gaps or artifacts. To ensure measurement accuracy, only the largest contiguous region within the mask was preserved, delineating the FAZ boundary for subsequent quantification of FAZ metrics, including area, perimeter, and acircularity index.

**Figure 1:**
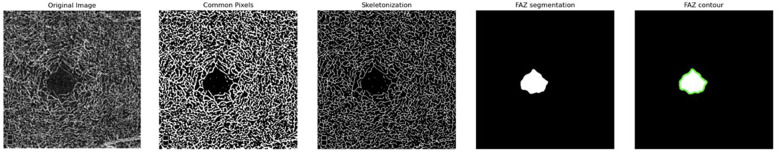
Post-segmented images for metric extraction. Common pixels were used to calculate vessel density and average vessel caliber. Skeletonized image was used to calculate skeleton density and fractal dimensions. FAZ segmentation was used to calculate FAZ area, perimeter, and acircularity index.

**Figure 2:**
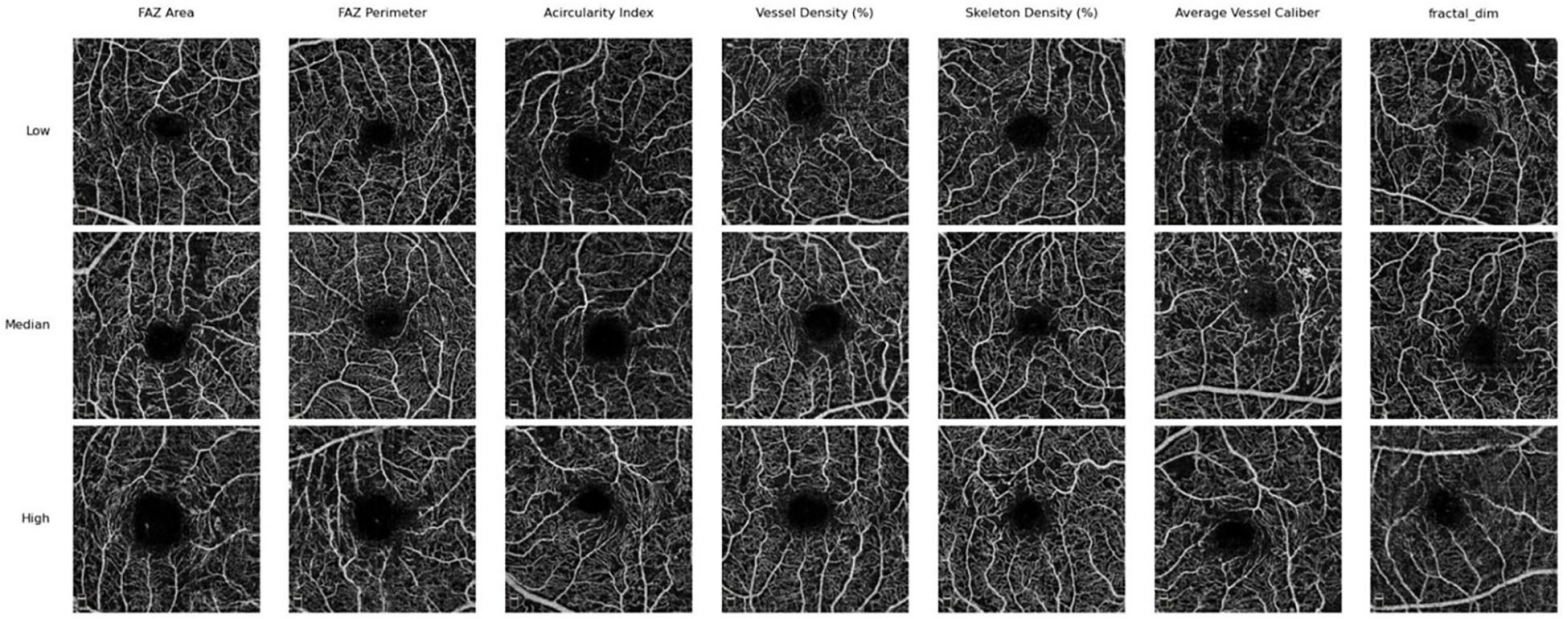
Representative en-face OCT-angiography slabs of the superficial vascular plexus illustrating the range of quantitative values observed in the cohort. Columns correspond to seven metrics analyzed in the study. Rows show eyes in the cohorts Low (<20^th^ percentile), Median (50^th^ percentile), and High (>80^th^ percentile) values for the metric in that column. In each column, the eye was selected to minimize Mahalanobis distance from the cohort median across all non-target metrics, ensuring that the primary visual distinction is driven by the illustrated parameter.

From the vessel segmentation and FAZ boundary, seven microvasculature indices were extracted from the deep and superficial layers of the OCTA images: acircularity index, average vessel caliber, FAZ area, FAZ perimeter, fractal dimensions, skeleton density, and vessel density (**Figure 1**). Definitions of these indices are listed below, and have been previously described and validated in literature (12, 13). Representative images of selected indices are shown in **Figure 2**.

1. Acircularity index: A dimensionless measure of irregularity and ischemia in the avascular region of the central fovea
2. Average vessel caliber (um): The average diameter of all vessels in scanned area
3. FAZ area (mm^2^): An area measurement of the central region of the fovea that lacks capillaries
4. FAZ perimeter (mm): A measurement of the central fovea’s FAZ perimeter
5. Fractal dimension: A mathematical measurement of the branching of retinal vasculature
6. Skeleton density (%): The percentage of the total retinal area covered by vessels, where each vessel is converted into a skeletonized line, and the total line length is calculated as a percentage of the retinal area.
7. Vessel density (%): The percentage of the total retinal area that is covered by vessels.

### Statistical analysis

**Figure 3:**
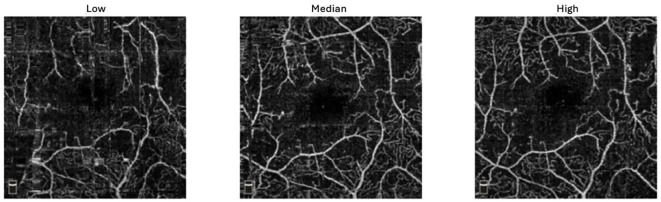
Representative en-face OCT-angiography slabs of the superficial vascular plexus with differing signal strengths. Figure shows images of low (signal strength = 3), median (signal strength = 6) and high-quality (signal strength = 9) images of the same patient. The images were taken within the same hour of each other and variations in quality was due to natural clinical process and not artificially induced.

Statistical analysis was performed using RStudio software (version 4.4.2). Baseline characteristics of patients in each DR category were compared using one-way analysis of variance (ANOVA) or Kruskal-Wallis tests for continuous variables, and Chi-square or Fischer’s exact tests for categorical variables, depending on data distribution. OCTA-derived microvascular metrics and visual acuity were analyzed at the eye level, whereas demographic variables were collected at the patient level. To reduce the variability of quantitative metrics, we screened OCTA images for signal strength prior to statistical analysis, and images with a signal strength of ≤ 5 were excluded from analysis. Representative images showing scans of different signal strengths are shown in **Figure 3**. For each patient visit, the mean value of each image with signal strength > 5 was used for subsequent analysis, as we find qualitatively that this is sufficient to reduce background noise and improve quantitative analysis (14).

To evaluate the progression of disease in terms of microvascular factors, linear regression analyses were performed to assess changes in each factor over time. Prior to analysis, OCTA-derived microvascular metrics were assessed for normality using the Shapiro-Wilk test. Metrics were approximately normally distributed, supporting the use of parametric statistical analyses. One-way ANOVA compared the slopes of the linear regressions between patients with different DR categories to evaluate the rate of progression in each factor. *Post-hoc* Tukey’s tests identified significant (*p* value ≤ 0.05) pairwise intergroup differences in the rate of change of vessel metrics for different classes. Specifically, the slopes of the linear regressions represent the estimated annual rate of change within each DR severity group, and ANOVA with *post-hoc* Tukey’s testing compared these within-group annual slopes across groups to identify which DR classes differ in their rate of longitudinal microvascular change.

To account for inclusion of both eyes from the same subject and the imbalanced distribution of DR severity, we additionally fit linear mixed-effects models with subject-level random intercepts. These models included time and DR severity as fixed effects. Although HbA1c was collected at baseline, longitudinal HbA1c measurements were not available. Therefore, HbA1c was not included as a covariate in the mixed-effects model. This analysis was performed excluding healthy controls to assess whether inclusion of healthy controls influenced statistical significance.

## Results

A total of 328 eyes from 164 adult patients with type II diabetes and 33 eyes from 17 healthy controls were included in this study. From the collected images, 18% of total scans were excluded due to poor signal strength and images from 7 eyes were excluded for lack of data availability. Images from a total of 55 eyes from patients with diabetes but no diabetic retinopathy (NDR), 7 eyes with mild NPDR, 216 eyes with moderate NPDR, 6 eyes with severe NPDR, 37 eyes with proliferative DR, and 33 eyes from healthy controls were selected for analysis, yielding a total of 321 eyes. Images from healthy controls were obtained every 12 months for 3 years. Baseline demographic data is shown in **Table 1**, and baseline OCTA quantitative metric values are shown in **Table 2**. We found significant differences across DR classes at baseline for HbA1c (p = 0.032), duration of diabetes (p = 0.021), and macular volume (p = 0.048) (**Table 1**). Additionally, the percentage of excluded scans significantly differed across groups (p < 0.001), with the highest rate of exclusion in the PDR cohort (24.7%), which likely reflects media opacity common in advanced disease.

**Table 1:** Baseline demographic characteristics. Continuous variables were compared across DR severity groups using one-way ANOVA or Kruskal-Wallis testing, as appropriate based on distribution.

	NDR	Mild NPDR	Moderate NPDR	Severe NPDR	PDR	p value
Number of patients	32	7	111	4	22	<0.001
HbA1c, % (Median [IQR])	6.70 [6.4-8.2]	7.3 [7.0-8.0]	7.7 [7.0-8.8]	7.9 [7.9-11.4]	7.6 [6.1-8.9]	0.032
Duration of Diabetes, years (Median [IQR])	15 [10-20]	26 [21-30]	20 [14-26]	17 [5-20]	22 [20-29]	0.021
Age, years (Median [IQR])	66 [62-71]	66 [65-70]	61 [54-69]	63 [63-64]	59 [54-64.5]	0.386
Sex, % male	43.7%	42.8%	53.4%	0.0%	50.4%	0.108
Eye level characteristics						
Number of eyes	55	7	216	6	37	<0.001
CSF Thickness (Median [IQR])	277.50 [258.00-295.75]	299.50 [294.75-329.00]	282.00 [257.25-314.00]	310.00 [268.00-313.00]	267.50 [249.25-323.25]	0.351
Macular Volume (Median [IQR])	8.36 [8.15-8.69]	8.56 [8.21-9.14]	8.64 [8.21-9.16]	8.58 [8.34-8.95]	8.29 [8.10-9.17]	0.048
Visual Acuity (logMAR, mean ± SD)	0.15 ± 0.19	0.04 ± 0.07	0.12 ± 0.32	0.47 ± 0.86	0.18 ± 0.20	0.213
Scans with DME	0 (0%)	2 (25%)	34 (18.38%)	1 (16.67%)	9 (25.00%)	0.188
% scans excluded	12.1	9.3	19.3	11.4	24.7	<0.001

Categorical variables were compared using chi-square or Fisher’s exact testing.

NDR, no diabetic retinopathy; IQR, interquartile range; NPDR, non-proliferative diabetic retinopathy; SD, standard deviation; PDR, proliferative diabetic retinopathy; DME, diabetic macular edema; HbA1c, hemoglobin A1c; CSF, central subfield thickness.

**Table 2:** Baseline OCTA characteristics in the deep and superficial retinal layers.

	Healthy controls (n = 33)	NDR (n = 55)	Mild NPDR (n = 7)	Moderate NPDR (n = 216)	Severe NPDR (n = 6)	PDR (n = 37)	P value
Superficial layer							
Acircularity	1.353 ± 0.160	1.552 ± 0.309	1.526 ± 0.273	1.897 ± 0.493	2.377 ± 0.473	2.181 ± 0.614	<0.001 *
Average Vessel Caliber (um)	22.303 ± 0.487	23.038 ± 0.651	23.296 ± 0.472	23.352 ± 1.115	24.172 ± 0.538	23.716 ± 1.144	<0.001 *
FAZ Area (mm2)	0.323 ± 0.084	0.376 ± 0.140	0.312 ± 0.109	0.501 ± 0.297	0.521 ± 0.169	0.623 ± 0.355	<0.001 *
FAZ Perimeter (mm)	2.721 ± 0.554	3.328 ± 1.020	2.988 ± 0.803	4.718 ± 2.300	6.040 ± 1.791	3.260 ± 2.181	<0.001 *
Fractal Dimension	1.763 ± 0.003	1.755 ± 0.008	1.754 ± 0.005	1.741 ± 0.019	1.738 ± 0.009	1.731 ± 0.021	<0.001 *
Skeleton Density (%)	15.867± 0.625	14.580 ± 1.095	14.207 ± 0.454	13.134 ± 1.715	12.606 ± 0.843	12.137 ± 1.629	<0.001 *
Vessel Density (%)	35.839± 1.090	33.969 ± 1.981	33.523 ± 0.841	30.924 ± 13.027	30.850 ± 1.724	29.022 ± 2.946	<0.001 *
Deep layer							
Acircularity	1.154 ± 0.0874	1.247 ± 0.283	1.303 ± 0.311	1.481 ± 0.460	1.448 ± 0.351	1.615 ± 0.482	<0.001 *
Average Vessel Caliber (um)	19.906 ± 0.282	20.409 ± 0.484	20.623 ± 0.431	21.189 ± 0.717	21.699 ± 0.522	21.855 ± 0.725	<0.001 *
FAZ Area (mm2)	0.200 ± 0.068	0.223 ± 0.139	0.190 ± 0.157	0.304 ± 0.237	0.261 ± 0.142	0.375 ± 0.280	<0.001 *
FAZ Perimeter (mm)	1.803 ± 0.348	2.038 ± 1.312	1.936 ± 1.414	2.796 ± 1.868	2.574 ± 1.297	4.419 ± 2.004	<0.001 *
Fractal Dimension	1.776 ± 0.002	1.773 ± 0.004	1.772 ± 0.004	1.766 ± 0.008	1.764 ± 0.006	1.759 ± 0.009	<0.001 *
Skeleton Density (%)	18.430 ± 0.686	17.663 ± 0.967	17.378 ± 0.993	16.167 ± 1.189	15.746 ± 1.016	14.952 ± 1.127	<0.001 *
Vessel Density (%)	37.163 ± 1.115	36.491 ± 1.382	36.278 ± 1.469	34.648 ± 1.881	34.592 ± 1.854	33.052 ± 1.838	<0.001 *

Data presented as mean ± standard deviation. n number reports number of eyes.

*NDR, no diabetic retinopathy; NPDR, non-proliferative diabetic retinopathy; PDR, proliferative diabetic retinopathy; FAZ, foveal avascular zone.

Asterisks indicate statistically significant p-values (p < 0.05) from one-way ANOVA.

Bold values indicate P values < 0.05

**Figure 4:**
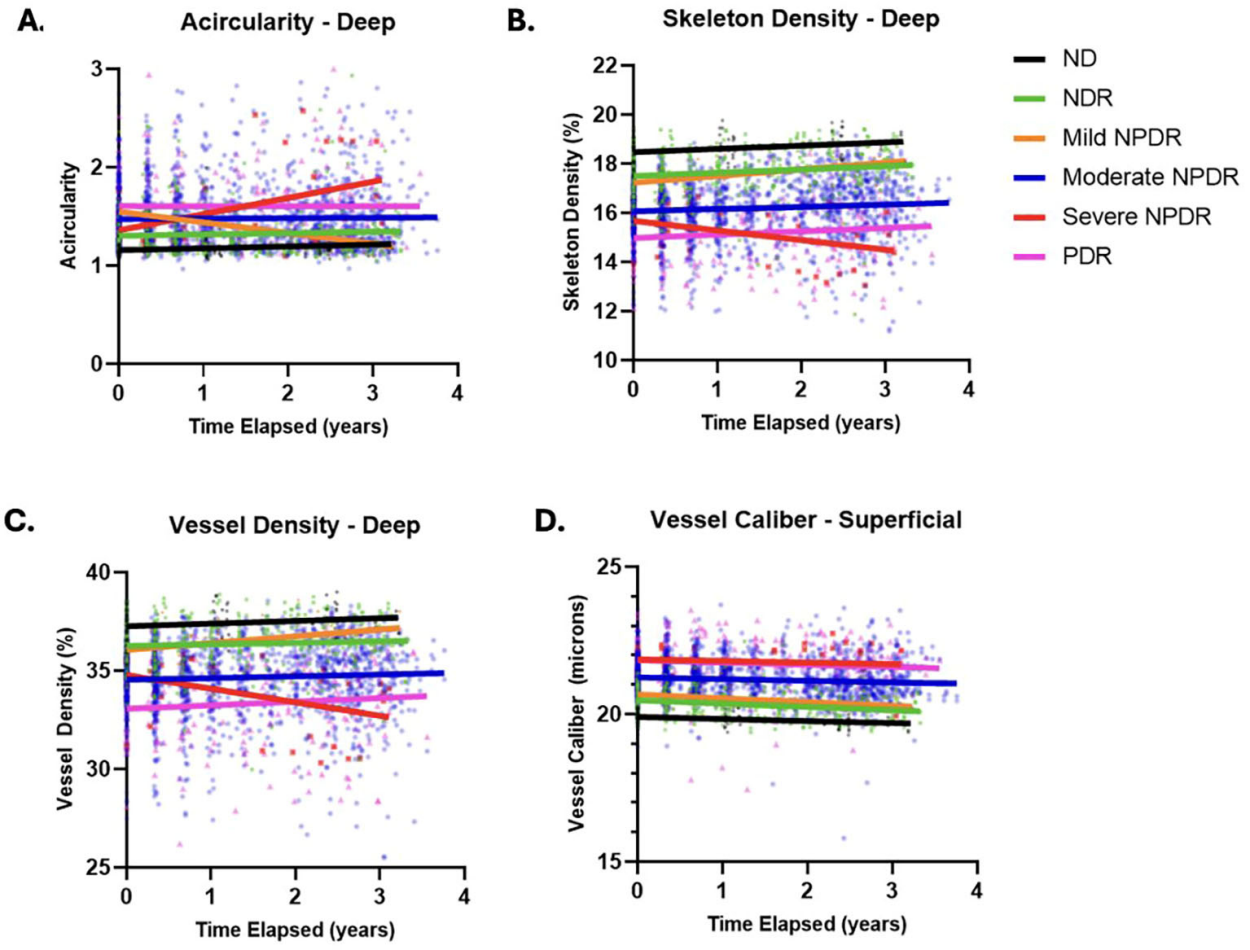
Change in microvasculature indices over time. Only biomarkers with statistically significant intergroup differences in annual rate of change are shown. **(A)** Deep layer acircularity index. **(B)** Deep layer skeleton density. **(C)** Deep layer vessel density. **(D)** Superficial layer average vessel caliber. DR classes are as follows: Non-diabetics (ND) (n = 33), diabetics with no diabetic retinopathy (NDR) (n = 55), Mild Non-Proliferative Diabetic Retinopathy (Mild NPDR) (n = 7), Moderate Non-Proliferative DR (Moderate NPDR) (n = 216), Severe Non-Proliferative DR (Severe NPDR) (n = 6), and Proliferative DR (PDR) (n = 37). Each data point represents an individual patient measurement, and the solid lines represent best-fit linear regression lines for each DR class.

**Figure 5:**
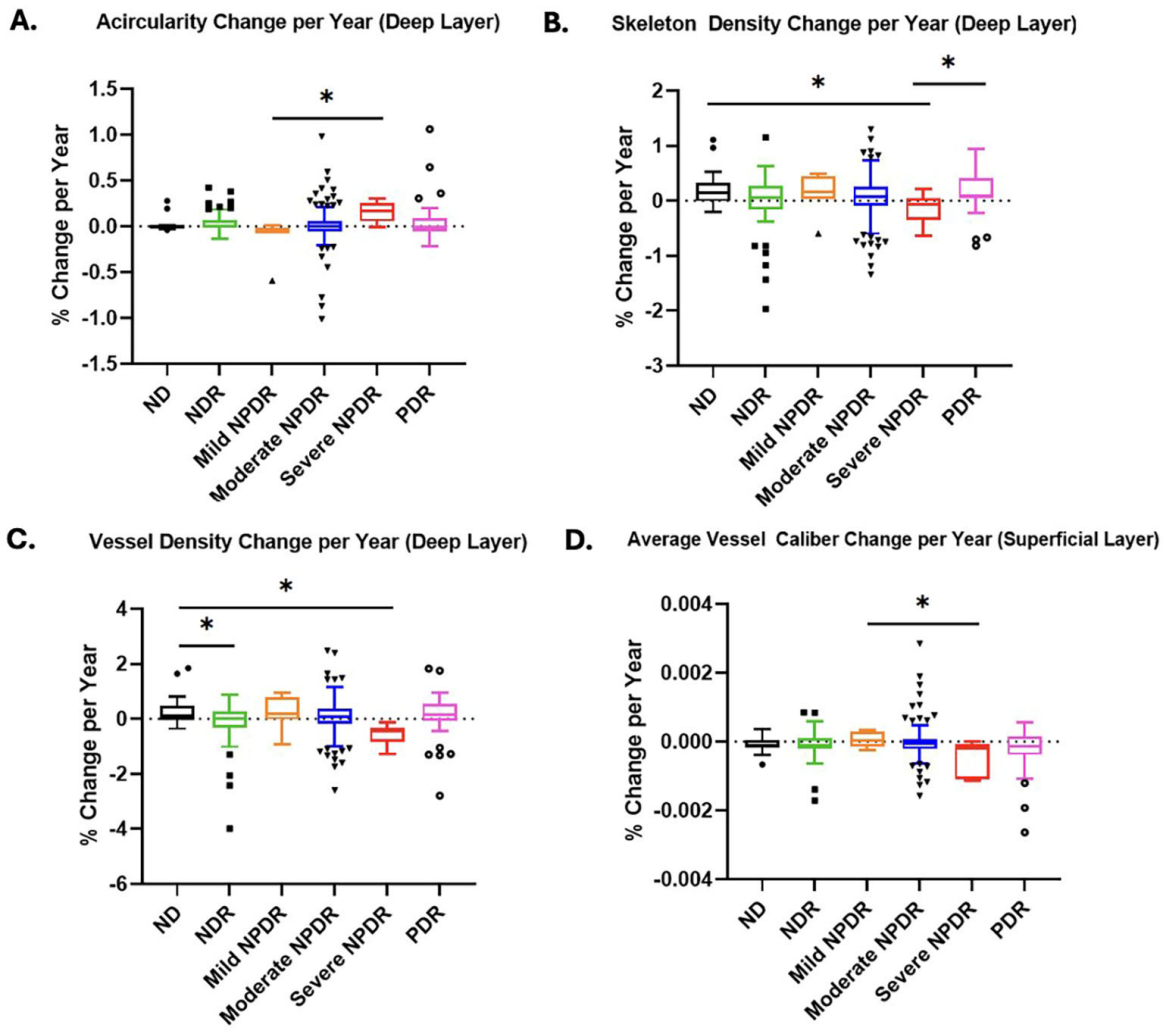
Average yearly change in microvasculature indices across DR classes. Only biomarkers with statistically significant intergroup differences in annual rate of change are shown. **(A)** Deep layer acircularity index. **(B)** Deep layer skeleton density. **(C)** Deep layer vessel density. **(D)** Superficial layer average vessel caliber. Boxplots show median and interquartile range (IQR). DR classes are as follows: Non-diabetics (ND) (n = 33), diabetics with no diabetic retinopathy (NDR) (n = 55), Mild Non-Proliferative Diabetic Retinopathy (Mild NPDR) (n = 7), Moderate Non-Proliferative DR (Moderate NPDR) (n = 216), Severe Non-Proliferative DR (Severe NPDR) (n = 6), and Proliferative DR (PDR) (n = 37). Asterisks indicate statistically significant differences (p < 0.05).

Linear regression analyses were performed to quantify the amount of change in each microvasculature index over time (**Supplementary Table 1**), and the slopes of the regression lines were used to compare the relative change in each parameter between DR categories (**Figures 4**, **5**). In the deep layer, intergroup comparison found a significant decrease in skeleton density (-0.296 ± 0.23% per year, p = 0.010) and vessel density (-0.563 ± 0.39% per year, p = 0.011) over time in patients with severe NPDR compared to healthy controls. Additionally, skeleton density showed a significant increase in patients with PDR (0.174 ± 0.37% per year, p = 0.010) compared to patients with severe NPDR. Acircularity index in the deep layer increased significantly in patients with severe NPDR (0.16 ± 0.12% per year, p = 0.045) compared to those with mild NPDR. In the superficial layer, the average loss of vessel caliber in patients with severe NPDR (-4.5e-4 ± 0.0005% per year, p = 0.040) was significantly greater than that of patients with mild NPDR, who on average gained 3.15e-2 microns in vessel caliber per year (**Figure 5**, **Table 3**).

**Table 3:** Vessel metrics percent change per year between DR classes. Data presented as mean ± standard deviation (% change per year).

	Healthy controls (n = 33)	NDR (n = 55)	Mild NPDR (n = 7)	Moderate NPDR (n = 216)	Severe NPDR (n = 6)		PDR (n = 37)	p value	Post-hoc
*Superficial Layer:*									
Acircularity	-0.007 ± 0.0346	0.035 ± 0.191	-0.007 ± 0.157	0.018 ± 0.157	-0.147 ± 0.126		0.003 ± 0.208	0.174	
Average Vessel Caliber (um)	-8.6e2 ± 0.02	-9.1e-2 ± 0.04	3.1e-2 ± 0.02	-3.6e-2 ± 0.04	-4.5e-1 ± 0.05		-2.3e-1 ± 0.06	0.040 *	Mild NPDR > Severe NPDR (p = 0.015)
FAZ Area (mm2)	-0.002 ± 0.009	-0.010 ± 0.076	-0.002 ± 0.010	0.0024 ± 0.086	-0.091 ± 0.068		0.006 ± 0.119	0.175	
FAZ Perimeter (mm)	-0.025 ± 0.098	0.001 ± 0.398	-0.030 ± 0.141	0.060 ± 0.790	-0.905 ± 0.742		0.036 ± 1.160	0.083	
Fractal Dimension	3.4e-4 ± 0.0008	-5.7e-4 ± 0.003	6.9e4 ± 0.001	-6.38e-5 ± 0.003	-5.1e-4 ± 0.002		7.7e-4 ± 0.003	0.444	
Skeleton Density (%)	0.161 ± 0.258	0.064 ± 0.358	0.073 ± 0.302	0.063 ± 0.533	0.398 ± 0.775		0.226 ± 0.751	0.325	
Vessel Density (%)	0.223 ± 0.357	0.011 ± 0.408	0.206 ± 0.505	0.109 ± 0.794	0.256 ± 1.127		0.198 ± 1.197	0.812	
*Deep layer:*									
Acircularity	0.011 ± 0.07	0.035 ± 0.11	-0.12 ± 0.21	0.007 ± 0.18	0.16 ± 0.12		0.05 ± 0.24	0.045 *	Severe NPDR > Mild NPDR (p = 0.026)
Average Vessel Caliber (um)	-7.1e-2 ± 0.12	-4.9e-2 ± 0.27	-2.7e-2 ± 0.15	-3.8e-2 ± 0.28	-0.15 ± 0.25		-0.18 ± 0.57	0.180	
FAZ Area (mm2)	-0.006 ± 0.012	-0.012 ± 0.100	-0.047 ± 0.141	0.002 ± 0.064	0.001 ± 0.056		0.021 ± 0.122	0.219	
FAZ Perimeter (mm)	-0.023 ± 0.058	-0.034 ± 0.603	-0.528 ± 1.442	0.025 ± 0.615	0.336 ± 0.550		0.239 ± 0.961	0.058	
Fractal Dimension	3.4e-4 ± 0.0008	-5.7e-4 ± 0.003	6.9e4 ± 0.001	-6.38e-5 ± 0.003	-5.1e-4 ± 0.002		7.7e-4 ± 0.003	0.723	
Skeleton Density (%)	0.203 ± 0.310	-0.025 ± 0.509	0.143 ± 0.362	0.066 ± 0.355	-0.296 ± 0.232		0.174 ± 0.371	0.010 *	Healthy controls > Severe NPDR (p = 0.046) PDR > Severe NPDR (p = 0.040)
Vessel Density (%)	0.276 ± 0.502	-0.151 ± 0.810	0.252 ± 0.624	0.066 ± 0.583	-0.563 ± 0.391	0.097 ± 0.839		0.011 *	Healthy controls > NDR (p = 0.039) Healthy controls > Severe NPDR (p = 0.008)

*NDR, no diabetic retinopathy.

NPDR, non-proliferative diabetic retinopathy.

PDR, proliferative diabetic retinopathy.

FAZ, foveal avascular zone.

Asterisks indicate statistically significant p-values from one-way ANOVA across all DR severity groups (p < 0.05). Tukey *post-hoc* analysis is reported for significant intergroup comparisons (p < 0.05).

Bold values indicate P values < 0.05.

In the mixed-effects analysis accounting for intra-subject correlation, DR severity remained significantly associated with longitudinal changes in multiple OCTA-derived microvascular metrics. Specifically, there were statistically significant differences across DR severity groups in vessel density in the superficial layer, as well as vessel density and skeleton density in the deep layer (**Supplementary Table 2**).

## Discussion

In this prospective longitudinal study of 361 eyes with repeated OCTA imaging over three years, we characterized rates of change in several retinal microvascular metrics across the spectrum of diabetic retinopathy severity. Overall, our findings demonstrate gradual but measurable longitudinal changes in retinal microvascular metrics, including vessel density, skeleton density, FAZ morphology, and vessel caliber. These trends were observed across the entire cohort and were not limited to a single disease stage, confirming that OCTA-derived biomarkers can capture subtle microvascular remodeling that accompanies diabetic retinopathy progression (18, 19). The magnitude of these changes suggests that microvascular remodeling in DR occurs slowly over time, with cumulative changes becoming detectable through repeated imaging. These findings highlight the potential utility of OCTA as a longitudinal monitoring tool, capable of identifying small but consistent trends in retinal microvascular structure that may precede clinically apparent progression.

Although several OCTA metrics differed across disease stages, the most pronounced longitudinal trends were observed in eyes with more advanced disease. Patients with severe NPDR showed a significant annual decrease in skeleton density and vessel density compared to healthy controls who demonstrated annual increases in these metrics. This reduction in capillary perfusion aligns with prior studies that have identified vessel density as a reliable anatomic marker of DR severity (20, 21). The observed difference in rates of vessel density loss between NPDR stages suggest that these changes may serve as early indicators of subtle disease progression, which has significant clinical implications for monitoring at-risk patients. Surprisingly, our study also found an increase in skeleton density in patients with PDR compared to severe NPDR, which may reflect compensatory revascularization or onset of neovascularization, a hallmark of advanced DR (22). However, it is important to interpret these observations cautiously, as the number of eyes with severe NPDR in our cohort was relatively small. Consequently, the broader significance of our findings lies less in the behavior of a single subgroup and more in the overall pattern of gradual microvascular change detected across the study population.

Significant changes in the FAZ, as measured by the acircularity index, provide insight into the progression of macular ischemia in DR and DME. Acircularity increased significantly in severe NPDR compared to mild NPDR, suggesting worsening ischemic damage as DR progresses leading to permanent visual impairment. This progressive irregularity in FAZ morphology may represent uneven capillary dropout along the FAZ margin, creating a more tortuous and irregular border as the disease advances. This observation is consistent with findings from longitudinal OCTA studies that report FAZ enlargement and irregularity as indicators of worsening microvascular dysfunction in DR (23, 24). The increasing acircularity without necessarily showing significant changes in FAZ area in the same timeframe suggests that border irregularity may be a more sensitive anatomic marker of early ischemic damage than simple FAZ enlargement. Clinically, this may explain why patients with DME do not regain full visual potential despite resolution of DME with anti-VEGF injections (25).

Our findings have important clinical implications. First, the identification of specific longitudinal markers, particularly deep plexus vessel and skeleton density, acircularity index, and superficial plexus vessel caliber, suggest that OCTA can be useful for monitoring of DR progression. Based on empirical observation from our cohort, in which the severe NPDR group demonstrated a mean annual skeleton density decline of -0.56% in the deep plexus, we hypothesize that. >0.5% annual decrease in deep plexus skeleton density may represent a threshold warranting closer monitoring. This threshold requires prospective validation in independent cohorts before clinical application. This may allow for earlier detection of high-risk patients and more timely intervention, potentially reducing the risk of vision-threatening complications. Moreover, the observed variability in OCTA metrics across different DR stages suggests that individualized monitoring strategies based on disease severity may be beneficial, with patients showing early changes in these metrics potentially requiring more frequent assessment than those with stable measurements. Our findings suggest that layer-specific analysis is essential, as the deep capillary plexus showed more consistent changes associated with disease progression than the superficial plexus, which aligns with the understanding that DR often affects deeper vessels first. Finally, while the present study assessed two-dimensional FAZ metrics (area, perimeter, acircularity index), volumetric FAZ analysis has emerged as a complementary three-dimensional parameter for evaluating DR severity (26). Future longitudinal studies should incorporate FAZ volume to provide depth-resolved quantification of macular ischemia.

While the present study focuses on longitudinal changes in retinal microvascular biomarkers, emerging evidence suggests that diabetic microvascular disease extends beyond the retinal circulation to involve the choroidal vasculature. Recent work demonstrates that eyes with diabetic retinopathy exhibit greater choriocapillaris hypoperfusion and impaired reperfusion following anti-VEGF therapy compared to non-diabetic eyes, highlighting the potential role for choroidal dysfunction in diabetic microvascular disease (27). Taken together with our findings of progressive retinal capillary rarefaction, these observations suggest that diabetic retinopathy may represent a more global ocular microangiopathy affecting both retinal and choroidal circulations. Future longitudinal studies incorporating simultaneous retinal and choroidal analyses may help clarify the interplay between these vascular beds and their relative contributions to disease progression.

Several limitations of our study must be acknowledged. The relatively small subset sample size, particularly in the severe NPDR group (n = 6), may limit the generalizability of our findings and requires validation in larger cohorts. These findings should be considered exploratory and interpreted with caution given the small sample size. The cohort size was defined by the parent INSPIRE trial protocol, and formal power calculations for individual OCTA endpoints were not performed as part of this study. Additionally, while we controlled for image quality by excluding images with signal strength ≤ 5, other potential confounding factors such as glycemic control fluctuations, blood pressure variation and medication changes during the follow-up period could influence microvascular metrics independent of DR progression. Our imaging processing pipeline, while systematic, may introduce measurement variability, particularly for smaller vessels near the resolution limit of the OCTA technology we used. Missing data points may have influenced our ability to detect subtle changes in OCTA metrics, particularly in the PDR group where clinical interventions could have altered the natural disease course. The treatments administered during follow-up, including anti-VEGF therapy and panretinal photocoagulation, were not systematically incorporated into the analyses and may have influenced OCTA metrics. Therefore, some of the longitudinal microvascular changes observed may reflect treatment effects rather than disease progression alone. Given the limited sample size, particularly within treatment subgroups, treatment-adjusted or treatment-excluded analyses were not feasible and should be explored in future studies. Additionally, because both DR severity and HbA1c were measured only at baseline and not reassessed longitudinally, progression status and glycemic control could not be reliably determined in this cohort. As a result, residual confounding related to time-varying glycemic control cannot be excluded. Additionally, the present analysis evaluates longitudinal OCTA change across baseline DR severity rather than direct OCTA correlates of clinically observed DR progression. Future longitudinal studies incorporating serial DR grading will be essential to compare OCTA biomarker trajectories between progressors and non-progressors.

An important consideration in interpreting these findings is the small magnitude of the longitudinal regression coefficients observed for many OCTA metrics. Annual changes on the order of 10–^4^ to 10–^3^ units could reflect expected variability associated with OCTA measurement. While the statistical significance of these changes is likely influenced by the large number of repeated measurements in this prospective dataset, the consistent directional trends observed across multiple metrics and disease stages suggest that these signals reflect gradual biologic remodeling rather than random measurement variability alone. This study demonstrates that OCTA may detect small but consistent longitudinal trends across repeated imaging sessions to provide meaningful insight into disease evolution.

In conclusion, our 3-year longitudinal study provides novel insight into the dynamic changes of retinal microvasculature across the spectrum of DR severity. By identifying specific OCTA biomarkers that correlate with disease progression, we provide a foundation for improved DR monitoring and early intervention strategies using a safe, non-invasive, and rapid imaging modality. Future research should focus on validating these anatomic markers in larger cohorts, establishing normative databases of progression rates, and determining whether interventions targeted at patients showing accelerated changes in these metrics can prevent vision-threatening complications. Continued research and longitudinal studies are essential to further validate these anatomic biomarkers and establish standardized protocols that can be integrated into clinical practice.

## Data Availability

The raw data supporting the conclusions of this article will be made available by the authors, without undue reservation.
