# Supplementary material for: Longitudinal changes in anatomic biomarkers on optical coherence tomography angiography in diabetic retinopathy
Source: Front Ophthalmol (Lausanne). 2026 Jul 9;6:1860036. doi: 10.3389/fopht.2026.1860036 (PMC13391325; doi:10.3389/fopht.2026.1860036)
Supplement: Supplementary file 1 [file DataSheet1.pdf]

Supplementary Data

Supplementary Table S1: Longitudinal mixed-effects model results for OCTA microvasculature metrics across DR severity groups. Models include subject-level random intercepts to account for repeated measurements and inclusion of both eyes from the same participant. Fixed effects include time and DR severity.

	Estimate	*Standard Error*	*Statistic*	*df*	*p value*
Superficial Layer:					
Acircularity	-1.06e-03	1.31e-02	-0.081	109.33	0.936
Average Vessel Caliber	3.64e-06	2.82e-05	0.129	295.00	0.897
FAZ Area	-3.00e-04	4.54e-03	-0.066	169.95	0.947
FAZ Perimeter	-7.91e-03	4.75e-02	-0.166	295.00	0.867
Fractal Dimensions	4.95e-04	3.42e-4	1.448	295.00	0.149
Skeleton Density	4.68e-02	2.95e-02	1.584	295.00	0.868
Vessel Density	1.47e-01	5.60e-02	2.624	141.45	**0.010 ***
Deep layer:					
Acircularity	-6.68e-04	8.56e-03	-0.08	187.51	0.938
Average Vessel Caliber	-1.35e-05	1.62e-05	-0.833	224.01	0.405
FAZ Area	7.10e-03	4.09e-03	1.735	188.44	0.084
FAZ Perimeter	4.66e-02	3.10e-02	1.503	197.17	0.134
Fractal Dimension	1.25e-04	1.51e-04	0.831	200.82	0.407
Skeleton Density	4.65e-02	2.35e-02	1.979	222.64	**0.049 ***
Vessel Density	8.34e-02	3.82e-02	2.183	196.30	**0.030 ***

*DR = diabetic retinopathy

df = degrees of freedom

FAZ = foveal avascular zone

Asterisks indicate statistically significant p values ($p < 0.05$)

Supplemental Table S2 – Longitudinal linear regression estimates of annual change in OCTA microvascular metrics by diabetic retinopathy severity.

	Healthy controls		*NDR*		*Mild NPDR*		*Moderate NPDR*		*Severe NPDR*		*PDR*	
Superficial Layer:	Estimate	SE	Estimate	SE	Estimate	SE	Estimate	SE	Estimate	SE	Estimate	SE
Acircularity	-5.62e-3	1.35e-2	-2.62e-3	1.58e-2	-6.15e-3	3.79e-2	-4.66e-3	1.07e-2	-1.44e-1	5.29e-2	-8.18e-4	2.99e-2
Average Vessel Caliber	-7.58e-5	4.16e-5	-1.02e-4	3.22e-5	-1.23e-4	6.03e-5	-5.21e-5	2.40e-5	-1.59e-3	5.77e-4	-7.14e-5	8.31e-5
FAZ Area	8.16e-3	8.50e-3	-7.50e-3	1.02e-2	-1.26e-2	1.69e-2	-3.38e-3	6.87e-3	-9.50e-2	2.48e-2	1.32e-2	1.97e-2
FAZ Perimeter	1.73e-2	5.02e-2	-4.54e-2	6.47e-2	-8.03e-2	1.32e-1	-2.14e-2	5.42e-2	-9.31e-1	2.25e-1	6.18e-2	1.69e-1
Fractal Dimensions	3.41e-5	2.97e-4	7.84e-4	4.58e-4	1.69e-3	7.05e-4	6.63e-4	4.48e-4	3.05e-3	1.73e-1	9.33e-4	1.22e-3
Skeleton Density	8.78e-2	5.08e-2	1.43e-1	5.44e-2	2.29e-1	7.61e-2	8.61e-2	3.95e-2	2.72e-1	1.73e-1	1.12e-1	1.06e-1
Vessel Density	7.59e-2	7.72e-2	1.90e-1	9.42e-2	3.52e-1	1.29e-1	1.41e-1	7.10e-2	1.18e-1	2.97e-1	2.10e-1	1.71e-1
Deep layer:												
Acircularity	1.83e-2	1.49e-2	1.31e-2	1.48e-2	-1.09e-1	7.24e-2	4.39e-3	8.46e-3	1.65e-1	5.99e-2	-6.85e-4	2.59e-2
Average Vessel Caliber	-6.89e-5	2.41e-5	-1.11e-4	2.60e-5	-1.28e-4	5.30e-5	-5.45e-5	1.74e-5	-5.23e-5	9.49e-5	-7.65e-5	5.63e-5
FAZ Area	5.18e-3	7.15e-3	-2.02e-3	1.04e-2	-4.90e-2	4.01e-2	2.26e-3	5.51e-3	6.89e-3	2.76e-2	2.20e-2	1.57e-2
FAZ Perimeter	3.57e-2	3.17e-2	4.81e-3	7.32e-2	-5.33e-1	4.15e-1	2.12e-2	3.61e-2	3.99e-1	2.75e-1	1.22e-1	1.21e-1
Fractal Dimension	1.14e-4	1.76e-4	7.26e-6	3.60e-4	1.09e-3	4.09e-4	1.36e-4	2.11e-4	-1.27e-3	1.01e-3	-5.87e-5	5.78e-4
Skeleton Density	1.33e-1	5.78e-2	1.33e-1	5.62e-2	2.71e-1	1.03e-1	8.99e-2	3.04e-2	-3.91e-1	1.54e-1	1.35e-1	7.79e-2
Vessel Density	1.37e-1	8.5e-2	7.34e-2	8.22e-2	3.46e-1	1.52e-1	8.85e-2	4.66e-2	-6.89e-1	2.55e-1	1.81e-1	1.29e-1

* NDR: no diabetic retinopathy

NPDR: non-proliferative diabetic retinopathy

PDR: proliferative diabetic retinopathy

FAZ: foveal avascular zone

SE: standard error

Estimate represents annual absolute change per year

Supplementary Table S3: Vessel metrics percent change per year between DR classes in images not screened for quality. Data presented as mean ± standard deviation (% change per year).

	Healthy controls	*NDR*	*Mild NPDR*	*Moderate NPDR*	*Severe NPDR*	*PDR*	*p value*
Superficial Layer:							
Acircularity	-0.007 ± 0.0346	0.029 ± 0.0159	-0.007 ± 0.045	0.019 ± 0.179	-0.147 ± 0.120	-2.5e-4 ± 0.206	0.239
Average Vessel Caliber	-8.6e-2 ± 0.214	-7.9e-2 ± 0.324	-3.2e-2 ± 0.218	-5.8e-2 ± 0.377	-0.116 ± 0.179	-5.1e-2 ± 0 .431	**0.021 ***
FAZ Area	-0.002 ± 0.012	-0.012 ± 0.043	-0.002 ± 0.010	0.006 ± 0.094	-0.093 ± 0.066	0.025 ± 0.146	0.058
FAZ Perimeter	-0.025 ± 0.098	-8.7e-4 ± 0.440	-0.028 ± 0.140	0.095 ± 0.932	-0.917 ± 0.702	0.153 ± 1.370	0.123
Fractal Dimensions	4.4e-4 ± 0.0006	2.7e-4 ± 0.002	5.2e4 ± 0.002	8.7e-4 ± 0.006	-3.8e-4 ± 0.007	2.3e-4 ± 0.008	0.728
Skeleton Density	0.161 ± 0.258	-0.050 ± 0.375	0.062 ± 0.285	0.119 ± 0.492	0.398 ± 0.727	0.100 ± 0.690	0.633
Vessel Density	0.223 ± 0.357	-0.008 ± 0.561	0.183 ± 0.472	0.208 ± 0.939	0.281 ± 1.060	0.145 ± 1.183	0.722
Deep layer:							
Acircularity	0.011±0.065	0.024 ± 0.100	-0.130 ± 0.221	0.019 ± 0.176	0.168 ± 0.120	-0.016 ± 0.169	**0.023 ***
Average Vessel Caliber	-7.1e-2 ± 0.115	-2.6e-2 ± 0.278	-2.3e-2 ± 0.144	-4.1e-2 ± 0.276	-0.152 ± 0.252	-5.9e-2 ± 0.320	0.895
FAZ Area	-0.006 ± 0.012	-0.016 ± 0.052	-0.050 ± 0.149	0.002 ± 0.078	-0.0005 ± 0.057	0.016 ± 0.093	0.179
FAZ Perimeter	-0.023 ± 0.058	-0.062 ± 0.288	-0.567 ± 1.525	0.044 ± 0.573	0.351 ± 0.560	0.084 ± 0.550	**0.028 ***
Fractal Dimension	3.4e-4 ± 0.0008	-4.1e-4 ± 0.002	6.9e4 ± 0.001	4.3e-5 ± 0.003	-5.7e-4 ± 0.002	2.3e-5 ± 0.004	0.757
Skeleton Density	0.203 ± 0.310	-0.028 ± 0.561	0.140 ± 0.358	0.140 ± 0.358	-0.306 ± 0.233	0.157 ± 0.449	**0.011 ***
Vessel Density	0.276 ± 0.502	-0.123 ± 0.783	0.252 ± 0.624	0.076 ± 0.629	-0.584 ± 0.415	1.001 ± 0.457	**0.022 ***

* NDR: no diabetic retinopathy

NPDR: non-proliferative diabetic retinopathy

PDR: proliferative diabetic retinopathy

FAZ: foveal avascular zone

Asterisks indicate statistically significant p-values ($p < 0.05$)
